# Complete Genome Sequence of the Endophytic Biocontrol Strain *Bacillus velezensis* CC09

**DOI:** 10.1128/genomeA.01048-16

**Published:** 2016-09-29

**Authors:** Xunchao Cai, Xingxing Kang, Huan Xi, Changhong Liu, Yarong Xue

**Affiliations:** State Key Laboratory of Pharmaceutical Biotechnology, School of Life Sciences, Nanjing University, Nanjing, China

## Abstract

*Bacillus velezensis* is a heterotypic synonym of *B. methylotrophicus*, *B. amyloliquefaciens* subsp. *plantarum*, and *Bacillus oryzicola*, and has been used to control plant fungal diseases. In order to fully understand the genetic basis of antimicrobial capacities, we did a complete genome sequencing of the endophytic *B. velezensis* strain CC09*.* Genes tightly associated with biocontrol ability, including nonribosomal peptide synthetases, polyketide synthetases, iron acquisition, colonization, and volatile organic compound synthesis were identified in the genome.

## GENOME ANNOUNCEMENT

*Bacillus velezensis* was first described by Wang et al. in 2008 (1) as a heterotypic synonym of *B. amyloliquefaciens*. Recently, *B. methylotrophicus*, *B. amyloliquefaciens* subsp. *plantarum*, and *B. oryzicola* were reclassified as heterotypic synonyms of *B. velezensis* based on comparative genomics and DNA-DNA relatedness calculations (2). The distinctive characteristics of *B. velezensis* include methanol utilization, plant-growth promotion, biocontrol of phytopathogens, and induced systemic resistance of the host (2–4). Many *B. velezensis* strains are used as plant-growth promoters and antagonists of plant pathogens in agriculture performances, and some of them have already been commercialized for increasing crop yield (2, 5–7).

*B. velezensis* CC09 was isolated from *Cinnamomum camphora* leaf tissue, which can be used to improve plant growth and prevent fungal diseases in plants caused by *Glomerella glycines*, *Rhizoctonia solani*, and *Alternaria alternate* by producing bioactive compounds (8, 9). Here, we report the complete genome of strain CC09, to better understand the expression, regulation, and transportation of proteins related to iturin A biosynthesis and the properties of other biocontrol behaviors.

Genomic DNA was extracted using the DNAiso reagent kit (TaKaRa, Japan), and a 250-bp paired-end insert library was sequenced on an Illumina MiSeq platform. The sequences were quality-filtered, resulting in 393 Mbp comprising 1,572,192 high-quality reads and representing a 94-fold coverage of the genome. The high-quality reads were then assembled by SPAdes assembler (10) to generate 20 contigs (≥500 bp; total length, 4,134,483 bp; *N*_50_ length, 690,639 bp). The obtained contigs were ordered into a draft genome with gaps (11), which was further defined by high-fidelity PCR (TaKaRa Prime STAR) to construct the complete genome. The genome size was 4,167,153 bp with an average GC content of 46.1%. A total of 4,021 coding sequences (CDSs) and 97 structural RNAs (73 tRNAs) were predicted by rapid annotation using PGAAP (genomes at http://ncbi.nlm.nih.gov) (12). Among the CDSs, 3,882 (96.54% of total) were protein-coding sequences, of which 2,143 (53.29%) were distributed over 26 functional gene categories in RAST (13).

Moreover, a total of 340.05 kb CDSs in the genome of CC09 (8.2% of the genome) encoding the proteins responsible for the synthesis of polyketide and nonribosomal peptides such as surfactin, iturin A, fengycin, bacillibactin, as well as genes associated with iron acquisition, colonization, and volatile organic compounds synthesis, which play important roles in the biocontrol processes of biocontrol agents like *B. velezensis* (2–4), were found in the genome of CC09. Without a doubt, these genome features make the endophytic strain CC09 an excellent candidate for biocontrol agents.

### Accession number(s).

This complete genome has been deposited at GenBank under the accession number CP015443. The strain has been deposited at the China Center of Industrial Culture Collection under the accession number CICC 24093.

## References

[1] Wang LT, Lee FL, Tai CJ, Kuo HP 2008 *Bacillus velezensis* is a later heterotypic synonym of *Bacillus amyloliquefaciens*. Int J Syst Evol Microbiol 58:671–675. doi:10.1099/ijs.0.65191-0. 18319476

[2] Dunlap CA, Kim SJ, Kwon SW, Rooney AP 2015 *Bacillus velezensis* is not a later heterotypic synonym of *Bacillus amyloliquefaciens*; *Bacillus methylotrophicus*, *Bacillus amyloliquefaciens* subsp. *plantarum* and ‘*Bacillus oryzicola*’ are later heterotypic synonyms of *Bacillus velezensis* based on phylogenomics. Int J Syst Evol Microbiol 66:1212–1217. doi:10.1099/ijsem.0.000858. 26702995

[3] Madhaiyan M, Poonguzhali S, Kwon SW, Sa TM 2010 *Bacillus methylotrophicus* sp. nov., a methanol-utilizing, plant-growth-promoting bacterium isolated from rice rhizosphere soil. Int J Syst Evol Microbiol 60:2490–2495. doi:10.1099/ijs.0.015487-0. 19966000

[4] Borriss R, Chen XH, Rueckert C, Blom J, Becker A, Baumgarth B, Junge H, Pukall R, Schumann P, Spröer C, Junge H, Vater J, Pühler A, Klenk HP 2011 Relationship of *Bacillus amyloliquefaciens* clades associated with strains DSM7^T^ and FZB42^T^: a proposal for *Bacillus amyloliquefaciens* subsp. *amyloliquefaciens* subsp. nov. and *Bacillus amyloliquefaciens* subsp. *plantarum* subsp. nov. based on complete genome sequence comparisons. Int J Syst Evol Microbiol 61:1786–1801. doi:10.1099/ijs.0.023267-0. 20817842

[5] Yao AV, Bochow H, Karimov S, Boturov U, Sanginboy S, Sharipov AK 2006 Effect of FZB 24® *Bacillus subtilis* as a biofertilizer on cotton yields in field tests. Arch Phytopathol Plant Protec 39:323–328. doi:10.1080/03235400600655347.

[6] Chen XH, Koumoutsi A, Scholz R, Eisenreich A, Schneider K, Heinemeyer I, Morgenstern B, Voss B, Hess WR, Reva O, Junge H, Voigt B, Jungblut PR, Vater J, Süssmuth R, Liesegang H, Strittmatter A, Gottschalk G, Borriss R 2007 Comparative analysis of the complete genome sequence of the plant growth-promoting bacterium *Bacillus amyloliquefaciens* FZB42. Nat Biotechnol 25:1007–1014. doi:10.1038/nbt1325. 17704766

[7] Chowdhury SP, Hartmann A, Gao X, Borriss R 2015 Biocontrol mechanism by root-associated *Bacillus amyloliquefaciens* FZB42—a review. Front Microbiol 6:780. doi:10.3389/fmicb.2015.00780. 26284057 PMC4517070

[8] Cai XC, Li H, Xue YR, Liu CH 2013 Study of endophytic *Bacillus amyloliquefaciens* CC09 and its antifungal cyclic lipopeptides. J Appl Biol Biotechnol 1:1–5. doi:10.7324/JABB.2013.1101.

[9] Yang HF, Xue YR, Yu XY, Liu CH 2014 Colonization of *Bacillus amyloliquefaciens* CC09 in wheat leaf and its biocontrol effect on powdery mildew disease. Microbiol China 30:481–488.

[10] Wattam AR, Abraham D, Dalay O, Disz TL, Driscoll T, Gabbard JL, Gillespie JJ, Gough R, Hix D, Kenyon R, Machi D, Mao C, Nordberg EK, Olson R, Overbeek R, Pusch GD, Shukla M, Schulman J, Stevens RL, Sullivan DE, Vonstein V, Warren A, Will R, Wilson MJC, Seung YH, Zhang C, Zhang Y, Sobral BW 2014 PATRIC, the bacterial bioinformatics database and analysis resource. Nucleic Acids Res 42:D581–D591. doi:10.1093/nar/gkt1099. 24225323 PMC3965095

[11] Darling AC, Mau B, Blattner FR, Perna NT 2004 Mauve: multiple alignment of conserved genomic sequence with rearrangements. Genome Res 14:1394–1403. doi:10.1101/gr.2289704. 15231754 PMC442156

[12] Angiuoli SV, Gussman A, Klimke W, Cochrane G, Field D, Garrity G, Kodira CD, Kyrpides N, Madupu R, Markowitz V, Tatusova T, Thomson N, White O 2008 Toward an online repository of standard operating procedures (SOPs) for (meta) genomic annotation. Omics 12:137–141. doi:10.1089/omi.2008.0017. 18416670 PMC3196215

[13] Aziz RK, Bartels D, Best AA, DeJongh M, Disz T, Edwards RA, Formsma K, Gerdes S, Glass EM, Kubal M, Meyer F, Olsen GJ, Olson R, Osterman AL, Overbeek RA, McNeil LK, Paarmann D, Paczian T, Parrello B, Pusch GD 2008 The RAST server: Rapid Annotations using Subsystems Technology. BMC Genomics 9:75. doi:10.1186/1471-2164-9-75. 18261238 PMC2265698

